# Causes of Microcephaly in the Zika Era in Argentina: A Retrospective Study

**DOI:** 10.1177/2333794X211040968

**Published:** 2021-08-20

**Authors:** Griselda Berberian, Rosa Bologna, María Guadalupe Pérez, Andrea Mangano, Marina Costa, Silvana Calligaris, María Alejandra Morales, Carlos Rugilo, Elisa Ruiz-Burga, Claire Thorne

**Affiliations:** 1 Hospital de Pediatria JP Garrahan, Buenos Aires, Argentina; 2 Instituto Nacional de Enfermedades Virales Humanas Dr. Julio I. Maiztegui, Buenos Aires Province, Argentina; 3 University College London, London, UK

**Keywords:** microcephaly, Zika, child health, birth defects, infectious disease

## Abstract

There are gaps in understanding the causes and consequences of microcephaly. This paper describes the epidemiological characteristics, clinical presentations, and etiologies of children presenting microcephaly during the Zika outbreak in Argentina. This observational retrospective study conducted in the pediatric hospital of Juan P. Garrahan reviewed the medical records of 40 children presenting microcephaly between March 2017 and November 2019. The majority (60%) were males and born full-term. At first evaluation, microcephaly was defined as congenital (31/40, 77%) and associated with other features (68%) such as seizures, developmental delay, non-progressive chronic encephalopathy, and West Syndrome. It was found manifestations restricted to central nervous system (55%), ocular (8/40, 20%), and acoustic (9/40, 23%) defects, and abnormal neuroimaging findings (31/39, 79%). Non-infectious diseases were the primary cause of isolated microcephaly (21/37, 57%), largely related to genetic diseases (13/21, 62%). Only 3 were children were diagnosed with Congenital Zika infection (3/16, 7.5%).

## Introduction

In 2015, an increase in the incidence of congenital microcephaly in newborns began to be observed in Brazil, with the initial ecological association with maternal Zika virus (ZIKV) infection later confirmed as causative.^1^ In Argentina, the first case of local transmission of ZIKV infection occurred in February 2016 in the province of Cordoba, followed by outbreaks in Tucumán, Salta, and Chaco. The first national case of congenital ZIKV syndrome (CZS) was reported in November 2016 in Tucumán.^2^ The National Network of Congenital Anomalies (RENAC) reported an increase in the birth prevalence of microcephaly in Argentina, from 4.1 per 10 000 in 2009 to 2015 to 6.9 per 10 000 in 2016/17, although this increase was substantially lower compared with other countries in the region like Brazil, Colombia and Venezuela.^2-4^ The last reported ZIKV case in Argentina was in early May, 2019. No new cases have subsequently been reported.^5^

Every year about 7.9 million (6%) infants born worldwide have major birth defects, with 3.3 million of these children estimated to die before reaching 5 years of age and 3.2 million surviving with a disability.^6^ The impact of birth defects is more critical in low and middle income countries (LMICs) where the conditions for prevention, treatment, and rehabilitation are challenging.^6^ In Latin America and the Caribbean (LAC), birth defects contribute up to 21% of mortality among children under 5 years.^7,8^ This burden has been complicated by the substantial number of newborns with microcephaly during the recent ZIKV outbreaks in the region.^9-12^ In Argentina, birth defects are the second most important cause of infant mortality and account for 28% of total infant deaths.^13^ As in many LMICs, the impact of birth defects nationally has been also observed in spontaneous abortion, comorbidity, high demand for medical and surgical treatments, social and emotional impact, and high economic costs.^14^

Microcephaly is a condition with multiple definitions and heterogeneous pathogenesis, and can be caused by a range of genetic and environmental factors that impact on the developing fetal brain.^15-18^ Prior to the re-emergence of ZIKV in 2016, it was well-established that some congenital infections such as *Toxoplasma gondii*, cytomegalovirus (CMV) and rubella, can result in microcephaly. However, the ZIKV pandemic highlighted gaps in both knowledge and surveillance of microcephaly around the world, including a lack of understanding with respect to the different causes and consequences of the condition.

In this context, we conducted a retrospective study of children with microcephaly evaluated at the pediatric Hospital Juan P. Garrahan, Buenos Aires during the period 2017 to 2019, when ZIKV outbreaks were ongoing, in order to describe the epidemiological, clinical, neuroimaging characteristics and etiologies overall and to examine any cases of CZS.

## Methods

This observational retrospective study was conducted through a review of medical records of all children younger than 3 years referred to the Infectious Diseases Clinic of the pediatric Hospital Juan P. Garrahan between March 2017 and November 2019, and diagnosed with microcephaly. Microcephaly was defined as the presence of a head circumference (HC) 2 standard deviations below the median for gestational age and sex^19^; it was further classified as congenital if it was first identified prenatally or at birth or as secondary if it occurred postnatally (ie, in an infant with a head circumference in the normal range at birth).^17^ The review of microcephaly cases during this time period was carried out in order to identify eligible children for inclusion in the ZIKAction Pediatric Registry of children with known ZIKV exposure in utero and/or with confirmed or suspected CZS. ZIKAction is an international consortium conducting interdisciplinary research on ZIKV epidemiology, natural history, and pathogenesis, with a particular emphasis on maternal and child health (www.zikaction.org). This study was reviewed and approved by the Comité Revisor de Investigacion, Ref: 962, Hospital de Pediatria Garrahan, Buenos Aires, Argentina. The parents or guardians provided written informed consent to enroll children in the study.

### Clinical and Laboratory Assessment

All clinical and laboratory evaluations were conducted as part of routine care, according to local guidelines on the diagnosis and assessment of children with microcephaly, which involves multidisciplinary input (ie, neurology, infectious diseases, radiology, ophthalmology). Thus, in addition to detailed medical history taking, neuroimaging, ophthalmological, and audiological evaluations were performed on all children. Neuroimaging evaluations (ultrasonography, Computed Axial Tomography, and/or Nuclear Magnetic Resonance) were classified as: presence of calcifications only; parenchymal compromise only; ventricular system compromise only; combinations of the above. All children underwent fundoscopy and had their hearing assessed with either otoacoustic emission (OAE) or auditory evoked potential testing, depending on their age. Karyotype and comparative hybridization array (array-CGH) were carried out when the geneticist considered that they were indicated.

Blood and/or urine samples from the children and their mothers were tested for toxoplasmosis, rubella, herpes simplex, Chagas disease, syphilis, CMV, and ZIKV. For ZIKV, real-time polymerase chain reaction (RT-PCR), and antigen-specific Immunoglobulin M (MAC-ELISA) testing was conducted at the Hospital Juan P. Garrahan, with plaque reduction neutralization test (PRNT90) at the Instituto Nacional de Enfermedades Virales Humanas “Dr. Julio I. Maiztegui.”

In this manner, the causes of microcephaly such as genetic, toxic and metabolic disorders were studied. The final classification of the microcephaly cases was carried out according to the etiology (infectious or non-infectious).

### Data Collection and Analysis

Medical record review of all eligible children was conducted, to collect data on demographic, clinical, neuroimaging, and laboratory data as well as pregnancy and perinatal information. The data collected was processed using Epi-Info 7.2. Continuous variables were reported as median, range or interquartile range (IQR). Comparisons of variables were assessed using the chi-squared, Fisher’s exact test, or Rank sum test. A *P* value <.*05* was considered statistically significant.

## Results

Forty children younger than 3 years of age with microcephaly were included during the study period, of whom most (60%) were male (Table 1). The majority of children had been born in Argentina (27 in Buenos Aires, 7 in other provinces), with 6 (15%) born in other LAC countries; 10% were born preterm and 25% had a low birthweight (Table 1).

**Table 1.** Infant and Maternal Characteristics of Microcephaly Cases, N = 40.

Characteristic	n (%) or median (IQR)
Sex	
Male	24 (60)
Female	16 (40)
Country of birth	
Argentina	34 (85)
Venezuela	3 (7.5)
Bolivia	3 (7.5)
Gestational age, median (weeks)	38 (26-41)
<37 completed weeks	4 (10)
≥37 completed weeks	36 (90)
Birthweight, median (g)	2800 (1400-3640)
<2500 g	10 (25)
≥2500 g	30 (75)
Maternal age at delivery, median (years)	26 (22-31)
Microcephaly classification	
Congenital	31 (77.5)
Secondary/postnatal	9 (22.5)

### Initial Presentation

The median age at first evaluation at the Hospital Juan P. Garrahan was 6 months (IQR 2–31.5 months). Microcephaly was congenital in 31 (77%) children, while in 9 (23%) it developed postnatally. Microcephaly was the only abnormal presentation at the initial consultation for 13 patients (33%), while the remaining 27 children presented in association with other features, most commonly seizures (N: 12, 44%), developmental delay (N: 10, 37%), non-progressive chronic encephalopathy (N: 6, 22%), and West Syndrome (N: 8, 30%). The presenting clinical features of the children stratified by congenital or secondary microcephaly are presented in Table 2. Subsequently, 5 of the 13 children referred with isolated microcephaly were identified as having other abnormalities following additional investigations.

**Table 2.** Clinical Features and Causes of Microcephaly Cases, by Presentation.

	Congenital microcephaly	Secondary microcephaly	Total
	N = 31, n (%)	N = 9, n (%)	N = 40 n (%)
Neurological/neurodevelopmental diagnoses			
Seizures	12 (38.7)	0	12 (30.0)
West syndrome	6 (19.4)	2 (22.2)	8 (20.0)
Chronic encephalopathy	4 (12.9)	2 (22.2)	6 (15.0)
Intellectual disability	9 (29.0)	1 (11.1)	10 (25.0)
Vision and hearing impairment			
Ocular abnormality	8 (25.8)	0	8 (20.0)
Sensorineural deafness	6 (19.4)	3 (33.3)	9 (22.5)
Other clinical features			
Congenital heart disease	2	1	3
Cleft palate	1	0	1
Arthrogryposis	1	0	1
Hepatomegaly	2	0	2
Hematological	1	0	1
Cause of microcephaly (n = 40)			
Genetic	11 (35.5)	2 (22.2)	13 (32.5)
Hypoxic-ischemic encephalopathy	3 (9.7)	3 (33.3)	6 (15.0)
Metabolic	2 (6.5)	0	2 (5.0)
Infectious	13 (41.9)	3 (33.3)	16 (40.0)
Unknown (lost to follow-up)	2 (6.5)	1 (11.1)	3 (7.5)

### Clinical and Radiological Investigations

Regarding clinical characteristics, for 22 (55%) children the pathologic manifestations were restricted to central nervous system (CNS) compromise, whilst the remaining 18 children had additional abnormalities identified (Table 2). Neuroimaging assessments were available for 39 children in total, and abnormal in 31 (79%): 7 children had combined parenchymal and ventricular abnormalities, 2 children had abnormalities in the 3 localizations, 10 showed intracranial calcifications only, 9 parenchymal abnormalities only, and 7 had ventricular system abnormalities only. Ocular abnormalities were identified in 8 children, all with congenital microcephaly: 5 had chorioretinitis, 1 papilla hypoplasia, 1 retinopathy with retinal fold and 1 leukocoria. Overall, 9 children had sensorineural deafness (1 unilateral, 8 bilateral), 3 of whom had secondary (postnatal) microcephaly; considering the child’s most severely affected ear, 1, 5, and 2 participants had mild, moderate, and severe hypoacusis respectively.

### Infection Investigations

Laboratory evidence of infectious diseases was recorded in 16 out of 37 cases (43%), with 9 (23%) children diagnosed with CMV, 4 (10%) with congenital toxoplasmosis, and 3 (7.5%) with CZS. The 3 cases of CZS (all male) were imported, with all 3 children born in Venezuela between May and June 2016; none of their mothers had laboratory confirmed ZIKV diagnosis whilst pregnant by PCR or serology, but were diagnosed with suspected ZIKV infection (2 mothers reported fever and rash during their pregnancy, and 1 fever and muscle/joint pain). The diagnoses of CZS were made based on clinical presentation and the epidemiological link in the context of exposure in a setting with circulating virus, with no laboratory evidence of ZIKV infection available in the medical records of the 3 children with CZS. All 3 mother-child pairs subsequently received serological testing after arrival in Argentina (>15 months after delivery in all cases), with 1 mother-child pair being IgG positive, 1 mother-child pair being IgG negative, and 1 being discordant (mother IgG positive and child IgG negative). In addition to congenital microcephaly, all 3 had intracranial calcifications, malformations of cortical development, and 1 child had chorioretinitis.

In addition to the 3 mothers of infants with CZS, one further mother reported fever and rash during her pregnancy (her infant’s microcephaly was found to be genetic in origin). It is also important to note that 1 child died because of CMV and neonatal complications unrelated to microcephaly.

### Causes of Microcephaly

The underlying cause of microcephaly was defined in the medical records of 37 children (93%), while 3 were lost to follow-up before a final diagnosis could be made. Overall, the etiology was classified as non-infectious for 21 children and infectious for 16. Pathogenesis was genetic in 13 cases, with the cause of the microcephaly determined to be due to hypoxic-ischemic encephalopathy in 6 cases and due to metabolic disease in 2. The distribution of causes by type of microcephaly (congenital and postnatal) is presented in Table 2.

Comparison of clinical characteristics between the non-infectious and infectious etiology groups showed that the former had significantly more cases with isolated microcephaly (eg, without ocular, auditory, or hematological abnormalities), at 67% (14/21) versus 31% (5/16) (*P* = .035), whilst there was a higher proportion of children with calcifications in the infectious etiology group compared with the non-infectious etiology group, at 50% (8/16) versus 14% (3/21) (*P* = .023 Fisher’s exact). No other significant differences were observed.

## Discussion

Our study, one of the first descriptive epidemiological investigations in Argentina of microcephaly following the emergence of ZIKV, provides a characterization of the patterns and etiologies of microcephaly in 40 children attending the pediatric hospital of J.P.Garrahan in Buenos Aires over a nearly 3 year period. The driver for this retrospective review of all microcephaly cases was a screening/surveillance process to identify cases of CZS for inclusion in the ZIKAction Pediatric Registry.

We found that microcephaly associated with other defects was more common than isolated microcephaly, similar to other studies^4^ and that the majority of cases had congenital microcephaly (77%). This differentiation is significant, as postnatal microcephaly is primarily associated with neuronal injury and cell death, while congenital microcephaly is related to fetal disruption of neuronal proliferation.^20^ It has therefore been suggested that postnatal microcephaly tends to have a more severe impact on neurodevelopmental function (particularly in the motor domain) than congenital onset^21^

Children with microcephaly in our study showed a broad spectrum of features, although presenting primarily with pathologic manifestations associated with CNS compromise, notably seizures, West Syndrome, and developmental delay. All children underwent neuroimaging and the majority (79%) had abnormalities detected—with parenchymal abnormalities dominating. In addition, 20% and 23% of our children with microcephaly had a diagnosis of vision impairment and sensorineural hearing impairment respectively, underscoring the complex needs that these children may present with, requiring multidisciplinary, long-term care. We noted the preponderance of males among the microcephaly cases in our study, which has been reported elsewhere,^22^ but not consistently.^23,24^

We were able to ascertain the probable cause of microcephaly for nearly all children following comprehensive investigations according to local guidelines. We found, consistent with other studies^16,19,21,24^, that there was a greater contribution of non-infectious than infectious etiologies within our case series, with genetic diseases a major cause in the former (accounting for 62% of cases) and causing microcephaly in a third of cases overall.

There were 3 different congenital infections identified as causes of microcephaly in our study, with CMV accounting for 56% of the infectious etiology group, and toxoplasmosis and ZIKV in the remaining cases. Prevalence of congenital CMV is estimated to range between 0.7% and 5% around the world, with higher prevalence in lower income countries,^17^ and is well recognized as a major cause of developmental disabilities. Whilst the majority of congenital CMV cases are asymptomatic at birth, evidence suggests that symptomatic presentation, particularly congenital microcephaly, is a strongly associated with adverse neurodevelopmental outcomes.^21,25,26^ In a review of 104 congenital microcephaly cases reported through the RENAC between April 2016 and March 2017, 73 were evaluated for congenital infections, with 5 cases attributed to ZIKV, 4 to CMV, 3 to toxoplasmosis, 2 to congenital Herpes simplex, and 1 to congenital syphilis.^4^

The 3 children with CZS (8% of all microcephaly cases) were all born in Venezuela in 2016 to mothers who reported symptoms consistent with ZIKV infection whilst pregnant, but without prenatal laboratory confirmation. Although we reviewed referrals of children with microcephaly during 2017 to 2019, it is not surprising that only a small proportion of the microcephaly cases were found to be due to congenital ZIKV infection, nor that these were imported cases only, given the hospital’s location in Buenos Aires, outside the tropical northern regions of Argentina where ZIKV outbreaks were occurring. The climatic and eco-epidemiological characteristics of Argentina explains why there were substantially fewer ZIKV cases compared to other countries in LAC, especially Brazil, Colombia, and Venezuela.^27^ However, considering the geographical proximity to higher burden countries, as well as population movements, clinicians nationally were alerted to the need to consider congenital ZIKV infection as a differential diagnosis for children with microcephaly or compatible pathology since 2016.

Evidence is still growing with respect to vertical transmission of ZIKV, which has been challenging to investigate due to factors including the high proportion of asymptomatic infections in pregnant women and challenges around testing and interpretation of potential laboratory markers of infection.^28,29^ A recent analysis using published data from 7 studies with prospective data on ZIKV in pregnancy provided preliminary estimates of average vertical transmission rates by trimester, respectively 47%, 28%, and 25% in the first, second and third trimesters.^30^ This same analysis estimated that probability of an infant having CZS symptoms was 9% following maternal ZIKV infection in the first trimester, and 3% and 1%, respectively where the mother was infected in the second and third trimesters. It is important to consider that 1 in 5 definite or probable cases of CZS are associated with brain abnormalities in infants without microcephaly, underscoring that surveillance should not focus solely on microcephaly.^31^ In our study, all 3 children with CZS had brain abnormalities (intracranial calcifications and malformations of cortical development), with chorioretinitis reported in 1. These clinical manifestations are consistent with the literature on defects associated with CZS,^32^ and confirm that CZS is a more complex spectrum of anomalies.^33,34^

This is one of the few published epidemiological studies of microcephaly in Argentina and provides a point of reference to demonstrate the diversity of characteristics that children with microcephaly can present, as well as the range of causes. Such understanding is important, considering that the prevalence of congenital microcephaly in Buenos Aires was estimated to be 1.8 per 10 000 (95% CI 1.3, 2.5) in 2010 to 2016.^35^ Microcephaly, particularly where it is accompanied by additional clinical features, can have serious implications for growth and development, with affected children at significant risk for delay across all aspects of development and for long-term disability.^21^

This study is limited by the retrospective design, with some missing data and loss to follow-up of a small number of cases. A strength of this single center study was that children were assessed in a homogenous manner, with all receiving neuroimaging, ophthalmological, audiological, and genetic evaluations as well as testing for congenital infections. The focus of this work was on identifying the potential causes of the microcephaly in the children referred to our hospital, as well as describing their clinical features at or shortly after presentation. It is therefore important to note that longer follow-up may identify additional adverse neurodevelopmental or other outcomes that may emerge over time, as well as providing important information on these children’s developmental trajectories.

This study contributes to improved understanding of the clinical presentation and causes of congenital and postnatal microcephaly in children in a LAC setting with limited ZIKV circulation. Over 3-quarters of the microcephaly cases in our study were congenital, and isolated microcephaly was present in only 20% of cases. Overall, non-infectious causes were the most frequent in children with microcephaly, but a high proportion (40%) were due to congenital infections. Congenital ZIKV infection was responsible for fewer than 10% of microcephaly cases, and all cases of CZS were born outside Argentina.

## References

[1] Rasmussen SA Jamieson DJ Honein MA Petersen LR. Zika virus and birth defects—reviewing the evidence for causality. New Engl J Med. 2016;374(20):1981-1987. doi:10.1056/NEJMsr1604338 27074377

[2] Pastrana A Albarracin M Hoffmann M, et al. Congenital Zika syndrome in Argentina: case series study [Sindrome de Zika congenito en la Argentina: presentacion de dos casos clinicos]. Arch Argent Pediatr. 2019;117(6):e635-e639. doi:10.5546/aap.2019.e635 31758900

[3] Kleber de Oliveira W Cortez-Escalante J De Oliveira WT, et al. Increase in reported prevalence of microcephaly in infants born to women living in areas with confirmed Zika virus transmission during the first trimester of pregnancy – Brazil, 2015. MMWR Morb Mortal Wkly Rep. 2016;65:242-247. 26963593 10.15585/mmwr.mm6509e2

[4] Tellechea AL Luppo V Morales MA, et al. Surveillance of microcephaly and selected brain anomalies in Argentina: relationship with Zika virus and other congenital infections. Birth Defects Res. 2018;110(12):1016-1026. doi:10.1002/bdr2.1347 29921033

[5] MSAL. Boletin Integrado de Vigilancia, Ministerio de Salud y Desarrollo Social - Presidencia de la Nacion, Vol. 450, SE 19. 2019.

[6] Christianson A Howson CP Modell B. Global Report on Birth Defects. The Hidden Toll of Dying and Disabled Children. New York: March of Dimes Birth Defects Foundation, 2006.

[7] Zarante I Hurtado-Villa P Walani SR, et al. A consensus statement on birth defects surveillance, prevention, and care in Latin America and the Caribbean. Rev Panam Salud Publica. 2019;43:e2. doi:10.26633/RPSP.2019.2 PMC6419921 31093226

[8] Liu L Oza S Hogan D, et al. Global, regional, and national causes of under-5 mortality in 2000–15: an updated systematic analysis with implications for the sustainable development goals. Lancet. 2016;388(10063):3027-3035. doi:10.1016/s0140-6736(16)31593-8 27839855 PMC5161777

[9] Metsky HC Matranga CB Wohl S, et al. Zika virus evolution and spread in the Americas. Nature. 2017;546(7658): 411-415. doi:10.1038/nature22402 28538734 PMC5563848

[10] Gardner LM Bóta A Gangavarapu K Kraemer MUG Grubaugh ND. Inferring the risk factors behind the geographical spread and transmission of Zika in the Americas. PLoS Negl Trop Dis. 2018;12(1):e0006194. doi:10.1371/journal.pntd.0006194 PMC5790294 29346387

[11] Larrandaburu M Luiz Vianna FS Anjos da Silva A Nacul L Vieira Sanseverino MT Schuler-Faccini L. Zika virus infection and congenital anomalies in the Americas: opportunities for regional action. Rev Panam Salud Publica. 2017;41:e174. doi:10.26633/RPSP.2017.174 PMC6645177 31384281

[12] Bautista LE Herrera VM. An assessment of public health surveillance of Zika virus infection and potentially associated outcomes in Latin America. BMC Public Health. 2018;18(1):656. doi:10.1186/s12889-018-5566-7 29793453 PMC5968501

[13] Dirección de Estadísticas e Información de Salud (DEIS). Estadísticas Vitales: Información Básica AÑO. 2016. http://www.deis.msal.gov.ar/index.php/indicadores-basicos/

[14] Bidondo MP Groisman B Barbero P Liascovich R. Public health approach to birth defects: the Argentine experience. J Community Genet. 2015;6(2):147-156. doi:10.1007/s12687-014-0209-x 25564015 PMC4356676

[15] Victora CG Schuler-Faccini L Matijasevich A Ribeiro E Pessoa A Barros FC. Microcephaly in Brazil: how to interpret reported numbers? Lancet. 2016;387(10019):621-624. doi:10.1016/s0140-6736(16)00273-7 26864961

[16] von der Hagen M Pivarcsi M Liebe J, et al. Diagnostic approach to microcephaly in childhood: a two-center study and review of the literature. Dev Med Child Neurol. 2014;56(8):732-741. doi:10.1111/dmcn.12425 24617602

[17] Devakumar D Bamford A Ferreira MU, et al. Infectious causes of microcephaly: epidemiology, pathogenesis, diagnosis, and management. Lancet Infect Dis. 2018;18(1):e1-e13. doi:10.1016/S1473-3099(17)30398-5 28844634

[18] Ashwal S Michelson D Plawner L Dobyns WB. Practice parameter: evaluation of the child with microcephaly (an evidence-based review): report of the quality standards subcommittee of the American Academy of Neurology and the practice committee of the Child Neurology Society. Neurology. 2009;73(11):887-897. doi:10.1212/wnl.0b013e3181b783f7 19752457 PMC2744281

[19] Arroyo HA. [Microcephaly]. Medicina. 2018;78 (Suppl 2):94-100. 30199373

[20] Passemard S Kaindl AM Verloes A. Microcephaly. Handb Clin Neurol. 2013;111:129-141. doi:10.1016/B978-0-444-52891-9.00013-0 23622158

[21] Gordon-Lipkin E Gentner MB German R Leppert ML. Neurodevelopmental outcomes in 22 children with microcephaly of different etiologies. J Child Neurol. 2017;32(9):804-809. doi:10.1177/0883073817707301 28482742

[22] Gallo LG Martinez-Cajas J Peixoto HM, et al. Another piece of the Zika puzzle: assessing the associated factors to microcephaly in a systematic review and meta-analysis. BMC Public Health. 2020;20:827. doi:10.1186/s12889-020-08946-5. Jun 1 2020;20(1):827. 32487247 10.1186/s12889-020-08946-5 PMC7266116

[23] Cragan JD Isenburg JL Parker SE, et al.; National Birth Defects Prevention Network. Population-based microcephaly surveillance in the United States, 2009 to 2013: an analysis of potential sources of variation. Birth Defects Res Part A Clin Mol Teratol. 2016;106(11):972-982. doi:10.1002/bdra.23587 PMC5485911 27891783

[24] Hansen M Armstrong PK Bower C Baynam GS. Prevalence of microcephaly in an Australian population-based birth defects register, 1980–2015. Med J Aust. 2017;206(8):351-356. 28446117 10.5694/mja16.00966

[25] Alarcon A Martinez-Biarge M Cabañas F Hernanz A Quero J Garcia-Alix A. Clinical, biochemical, and neuroimaging findings predict long-term neurodevelopmental outcome in symptomatic congenital cytomegalovirus infection. J Pediatr. 2013;163(3):828-34.e1. doi:10.1016/j.jpeds.2013.03.014 23587436

[26] Yamada H Tanimura K Tairaku S, et al. Clinical factor associated with congenital cytomegalovirus infection in pregnant women with non-primary infection. J Infect Chemother. 2018;24(9):702-706. doi:10.1016/j.jiac.2018.04.010 29735300

[27] Morris JK Dolk H Durán P Orioli IM. Use of infectious disease surveillance reports to monitor the Zika virus epidemic in Latin America and the Caribbean from 2015 to 2017: strengths and deficiencies. BMJ Open. 2020;10(12):e042869. doi:10.1136/bmjopen-2020-042869 PMC7735125 33310811

[28] Coelho AVC Crovella S. Microcephaly prevalence in infants born to Zika virus-infected women: a systematic review and meta-analysis. Int J Mol Sci. 2017;18(8):2017. doi:10.3390/ijms18081714 PMC5578104 28783051

[29] Ades AE Thorne C Soriano-Arandes A, et al. Researching Zika in pregnancy: lessons for global preparedness. Lancet Infect Dis. 2020;20(4):e61-e68. doi:10.1016/S1473-3099(20)30021-9 32085848

[30] Ades AE Soriano-Arandes A Alarcon A, et al. Vertical transmission of Zika virus and its outcomes: a Bayesian synthesis of prospective studies. Lancet Infect Dis. 2021;21(4):537-545. doi:10.1016/S1473-3099(20)30432-1 33068528 PMC7992034

[31] França GVA Schuler-Faccini L Oliveira WK, et al. Congenital Zika virus syndrome in Brazil: a case series of the first 1501 livebirths with complete investigation. Lancet. 2016;388(10047):891-897. doi:10.1016/s0140-6736(16)30902-3 27372398

[32] Moore CA Staples JE Dobyns WB, et al. Characterizing the pattern of anomalies in congenital Zika syndrome for pediatric clinicians. JAMA Pediatr. 2017;171(3):288-295. doi:10.1001/jamapediatrics.2016.3982 27812690 PMC5561417

[33] Oliveira Melo AS Malinger G Ximenes R Szejnfeld PO Alves Sampaio S Bispo de Filippis AM. Zika virus intrauterine infection causes fetal brain abnormality and microcephaly: tip of the iceberg? Ultrasound Obstet Gynecol. 2016;47(1):6-7. doi:10.1002/uog.15831 26731034

[34] Pomar L Musso D Malinger G Vouga M Panchaud A Baud D. Zika virus during pregnancy: from maternal exposure to congenital Zika virus syndrome. Prenat Diagn. 2019;39(6):420-430. doi:10.1002/pd.5446 30866073

[35] Bronberg R Groisman B Bidondo MP Barbero P Liascovich R. Birth prevalence of congenital anomalies in the City of Buenos Aires, Argentina, according to socioeconomic level. J Community Genet. 2020;11(3):303-311. doi:10.1007/s12687-019-00449-0 31900751 PMC7295923

